# Experimental infection of Korean native goats (*Capra aegagrus hircus*) with bovine viral diarrhea virus 1b

**DOI:** 10.1186/s12917-019-1955-0

**Published:** 2019-06-14

**Authors:** Jae-Ku Oem, Du-Gyeong Han, Kyoung-Seong Choi

**Affiliations:** 1 College of Veterinary Medicine, Chonbuk National University, Iksan, 54596 Republic of Korea; 2 Division of Bacterial Disease Research, Center for Infectious Disease Research, Korea National Institute of Health, Korea Centers for Disease Control and Prevention, Osong, 28159 Republic of Korea; 3 Department of Animal Science and Biotechnology, College of Ecology and Environmental Science, Kyungpook National University, Sangju, 37224 Republic of Korea

**Keywords:** Bovine viral diarrhea virus, Noncytopathic, Korean native goat, 5′-untranslated region, N-terminal protease region

## Abstract

**Background:**

Bovine viral diarrhea virus (BVDV) infects various ungulates and causes reproductive failure in infected goats. BVDV has been detected among goats in the Republic of Korea, but the route of transmission remains unclear. Here, we aimed to investigate whether BVDV-1b circulating among Korean cattle can be transmitted to Korean native goats (*Capra aegagrus hircus*) and characterize the outcomes of BVDV infection in these goats.

**Results:**

Four goats were inoculated intranasally with the Korean noncytopathic (ncp) BVDV-1b strain. Two goats exhibited clinical signs of illness, including coughing and nasal discharge. Nasal swabs and blood were collected to screen for viral RNA and BVDV antibodies. Using the 5′-untranslated region (UTR), viral RNA was detected in the nasal swabs of two goats (Goat 1 and 3) on 12 day post-inoculation (dpi) and in the blood sample of one goat (Goat 1) on 7 and 19 dpi. Using the N-terminal protease (N^pro^) region, viral RNA was detected in the blood sample of Goat 1 on 7 and 12 dpi. Antibodies to BVDV were detected in Goats 1 and 3 on 16–21 dpi using enzyme-linked immunosorbent assay. Sequence analysis of the virus from nasal swabs and blood samples, which was detected via RT-PCR, using the 5′-UTR and N^pro^ regions led to the identification of the strain as ncp BVDV-1b and revealed changes in the nucleotide sequence of these goats.

**Conclusions:**

Our results indicate that changes in the nucleotide sequence are associated with the establishment of BVDV infection in Korean native goats; these changes may be owing to a process required for the establishment of infection in a new host reservoir. Broadly, these findings highlight the importance of BVDV surveillance in ungulates other than cattle.

**Electronic supplementary material:**

The online version of this article (10.1186/s12917-019-1955-0) contains supplementary material, which is available to authorized users.

## Background

The genus *Pestivirus* within the family *Flaviviridae* comprises four species: border disease virus (BDV), bovine viral diarrhea virus (BVDV)-1, BVDV-2, and classical swine fever virus (CSFV). Other putative pestivirus species include Giraffe virus [1]; Pronghorn virus, isolated from a blind pronghorn antelope in the United States [2]; Bungowannah virus, detected in swine affected by reproductive failures including stillbirth and neonatal death in Australia [3]; and HoBi-like virus, also known as BVDV-3 or atypical pestivirus, and recognized as a bovine pathogen having a clinical presentation similar to that of BVDV-1 or BVDV-2 [4–6]. Unlike CSFV, BDV and BVDV are not host-specific and can infect a wide range of artiodactyls [7]. Transplacental infection of the fetus with pestivirus before the onset of immunological maturity leads to the birth of persistently infected animals, which are the main source of viral transmission [8–10].

BVDV can infect small ruminants, and clinical signs in these animals are similar to those in cattle [11]. Field cases of BVDV in goats are typically characterized by reproductive failure, including abortion and poor viability of neonates [12–14]. In a recent study conducted by our group, natural infection with BVDV was detected in Saanen goats [15]. None of the goats infected with BVDV exhibited clinical signs. However, the BVDV transmission route in these goats has not yet been identified. Because most goats raised in the Republic of Korea (ROK) share their habitats with cattle, it is possible that BVDV transmission frequency is higher among these animals.

The population of Korean native goats (*Capra aegagrus hircus*) has been increasing gradually in the ROK, and their main products are meat and milk. Few studies have reported BVDV infection in goats in the ROK [15, 16], and little is known about BVDV circulation and transmission in goat farms. Recently, we found that BVDV-1b is the predominant subtype circulating among Korean indigenous cattle [17]. Therefore, here, we aimed to investigate whether BVDV-1b infection can be established in Korean native goats via intranasal (IN) inoculation, evaluate the circulation of BVDV between cattle and Korean native goats, and characterize the outcome of BVDV infection in Korean native goats.

## Results

Of the four Korean native goats infected with BVDV via IN inoculation, two goats were symptomatic, exhibiting coughing and nasal discharge; one goat displayed respiratory signs with coughing on 2–5 days post-infection (dpi) and nasal discharge on 12–19 dpi. The respiratory signs were marked and persistent in this goat. On the other hand, the other goat exhibited only nasal discharge (Table 1); however, other clinical signs, including loss of appetite, diarrhea, and pyrexia, were not observed. Additionally, the remaining two goats showed no clinical symptoms until the end of this experiment. No clinical signs were observed in mock-infected animals. In this study, no other diagnostic assay for respiratory signs was performed, and the nasal discharge of the two goats was tested only for BVDV.

**Table 1:** Clinical signs of the Korean native goats after intranasal inoculation

Days post-infection (dpi)	Clinical signs			
	Goat 1	Goat 2	Goat 3	Goat 4
0	–	–	–	–
2	Coughing	–	–	–
5	Coughing	–	–	–
7	–	–	–	–
9	–	–	–	–
12	Nasal discharge	–	–	–
14	–	–	–	–
16	Coughing, nasal discharge	–	Nasal discharge	–
19	Nasal discharge	–	–	–
21	–	–	–	–

“-”; no clinical signs

Prior to inoculation, the goats were negative for BVDV antigen and antibody. Viral RNA was not detected in mock-infected animals. As shown in Table 2, using the 5′-untranslated region (UTR) gene, viral RNA was detected in the nasal swabs of two BVDV-inoculated goats on 12 dpi (Goats 1 and 3) and in the blood sample of one goat (Goat 1) on 7 and 19 dpi. Viral RNA was not detected in nasal swabs but was detected in the blood of Goat 1 on 7 and 12 dpi when primers against the N-terminal protease (N^pro^) region were used (Table 2). The mock-infected goats remained seronegative throughout the experiment. Two BVDV-inoculated goats (Goat 1 and 3) produced viral antibodies, whereas the other two goats remained seronegative during the experiment (Table 2).

**Table 2:** Viral RNA detection by RT-PCR of nasal swabs and blood, and the presence of anti-BVDV antibodies by ELISA in four Korean native goats following intranasal infection

Days post-infection (dpi)	Goat 1				Goat 2				Goat 3				Goat 4			
	RT-PCR			ELISA	RT-PCR			ELISA	RT-PCR			ELISA	RT-PCR			ELISA
	NS^a^	Blood		Serum	NS	Blood		Serum	NS	Blood		Serum	NS	Blood		Serum
	5′UTR	5′UTR	N^pro^		5′UTR	5′UTR	N^pro^		5′UTR	5′UTR	N^pro^		5′UTR	5′UTR	N^pro^	
0	–	–	–	–	–	–	–	–	–	–	–	–	–	–	–	–
2	–	–	–	–	–	–	–	–	–	–	–	–	–	–	–	–
5	–	–	–	–	–	–	–	–	–	–	–	–	–		–	–
7	–	+	+	–	–	–	–	–	–	–	–	–	–	–	–	–
9	–	–	–	–	–	–	–	–	–	–	–	–	–	–	–	–
12	+	–	+	–	–	–	–	–	+	–	–	–	–	–	–	–
14	–	–	–	–	–	–	–	–	–	–	–	–	–	–	–	–
16	–	–	–	+	–	–	–	–	–	–	–	+	–	–	–	–
19	–	+	–	+	–	–	–	–	–	–	–	+	–	–	–	–
21	–	–	–	+	–	–	–	–	–	–	–	+	–	–	–	–

^a^*NS* nasal swabs

“-”: not detected, “+”: detected

**Fig. 1:**
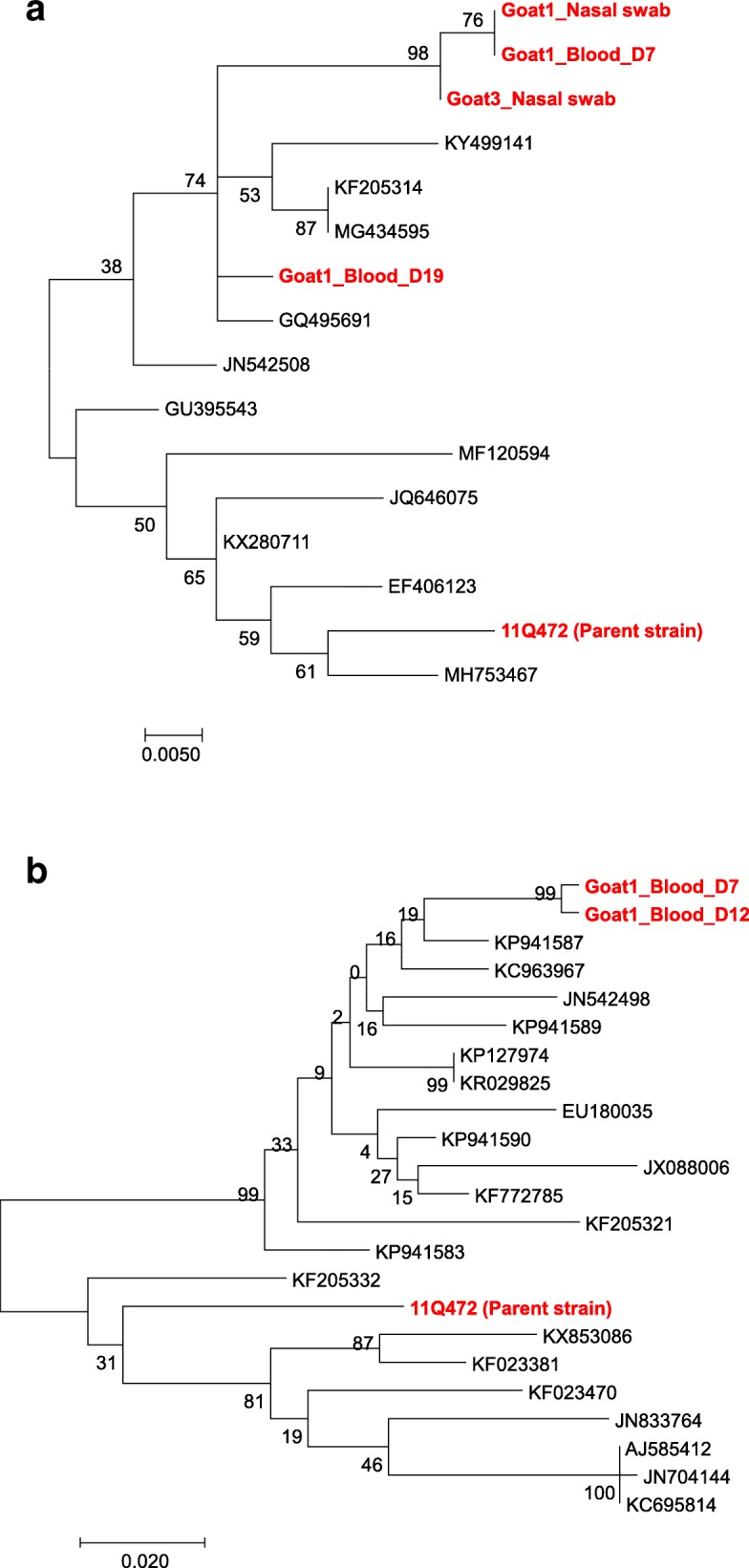
Phylogenetic tree based on 5′-UTR (288 bp) (**a**) and N^pro^ region (423 bp) (**b**) nucleotide sequences. The isolates sequenced in this study and reference BVDV strains/isolates were used to construct the phylogenetic tree by employing the neighbor-joining method using MEGA6 software. Bootstrap values are shown at branch nodes

**Fig. 2:**
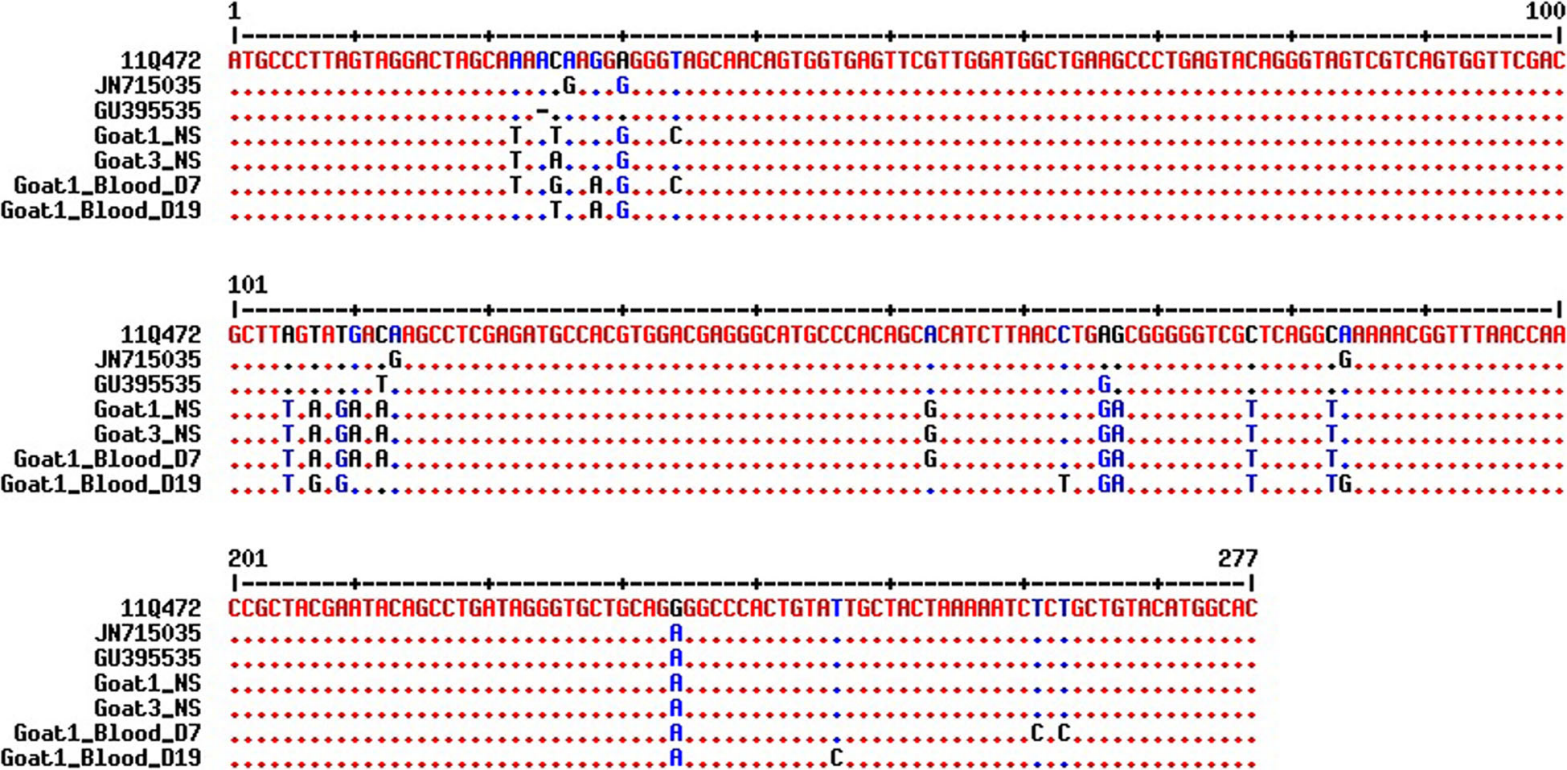
Nucleotide alignment of the 5′-UTR of BVDV. Nucleotide sequences from the nasal swabs (NS) of Goats 1 and 3 on 12 dpi and blood of Goat 1 on 7 and 19 dpi were determined and compared with the sequences of the parent strain (11Q472) and other cattle isolates (JN715035 and GU395535). Dots represent identical nucleotides

**Fig. 3:**
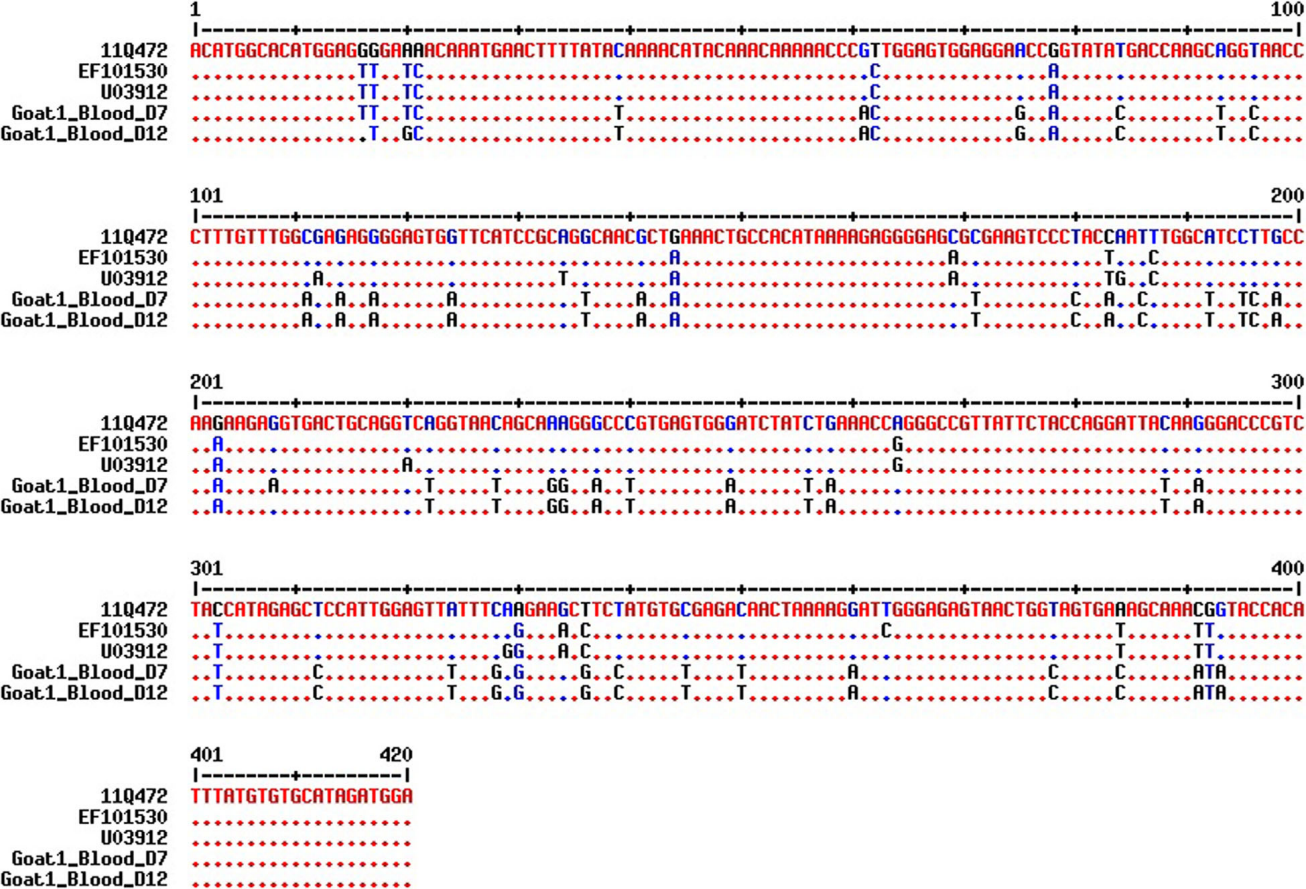
Nucleotide alignment of the N^pro^ region of BVDV. Nucleotide sequences from the blood of Goat 1 on 7 and 12 dpi were determined and compared with the sequences of the parent strain (11Q472) and other cattle isolates (EF101530 and U03912). Dots represent identical nucleotides

Nucleotide sequence analyses were conducted using both the 5′-UTR and N^pro^ regions to confirm BVDV infection in goats and compare the genomic sequences of isolates obtained from BVDV-inoculated goats and 11Q472 (parent strain) with those obtained from BVDV cattle isolates (Additional file 1: Table S1 and Additional file 2: Table S2). Phylogenetic analysis conducted based on 5′-UTR revealed that sequences obtained from the nasal swabs of two goats (Goats 1 and 3) and from the blood of Goat 1 on 7 and 19 dpi were those corresponding to ncp BVDV-1b. These three sequences (identified in the nasal swabs of Goats 1 and 3 and in the blood of Goat 1 on 7 dpi) belonged to the same clade, whereas the sequence obtained from blood on 19 dpi belonged to a separate clade (Fig. 1a). The phylogenetic tree of the N^pro^ region showed that the sequences amplified from the blood of Goat 1 on 7 and 12 dpi were those of ncp BVDV-1b, and these sequences were divergent from those of cattle isolates (Fig. 1b). As shown in Fig. 2 and Fig. 3, there were significant differences in the nucleotide sequences before (11Q472) and after inoculation. These differences in the nucleotide sequences were more frequent in the N^pro^ region than in the 5′-UTR (Fig. 3).

## Discussion

BVDV infection in small ruminants is similar to those in cattle. Postnatal infections cause mild clinical symptoms, including pyrexia and leukopenia, and infections in pregnant small ruminants may cause reproductive failures [11, 18–22]. Our findings revealed that experimental BVDV infection in Korean native goats via IN route showed a normal appearance with only mild respiratory signs such as coughing and nasal discharge and did not cause pyrexia, anorexia, and diarrhea. In a previous study, we reported BVDV infection in Saanen goats, which exhibited no clinical symptoms [15]. Several studies have demonstrated that clinical manifestation of BVDV infection in sheep and goats may differ depending upon inoculation route, age of the hosts, and the BVDV strain used [18–22]. Taken together, BVDV infection in Korean native goats induced via IN inoculation is accompanied only by respiratory symptoms. Further studies should focus toward investigating the clinical signs of BVDV infection in goats using various inoculation routes and viral strains.

In this study, viral RNA and serologic conversion were detected in only two of the inoculated goats, which exhibited coughing and nasal discharge. Two primers were used to detect BVDV infection in these goats, but PCR results were slightly different between the 5′-UTR and N^pro^ regions (Table 2). Although we could not conclude which gene is better for BVDV diagnosis, we suggest that both genes can be used for a more accurate diagnosis. Moreover, the serological response in goats was relatively different from that in cattle. Seroprevalence rates of 3–35% were detected in small ruminants, and they were relatively lower in goats than in sheep [20]. It is speculated that the antibody production varies between goats and sheep. In our previous study on calves, seroconversion was evident for less than a week after inoculation [23], whereas in the present study, BVDV-specific antibodies were produced by goats for approximately 2 weeks after inoculation (Table 2), indicating a host-specific difference in seroconversion. These results suggested that BVDV requires time to induce infection in a new host.

The present results revealed that the nucleotide sequences of both the 5′-UTR and N^pro^ regions observed in Korean native goats after inoculation were substantially different from those observed in cattle isolates (11Q472, JN715035, and GU395535 for 5′-UTR; EF101530 and U03912 for N^pro^). The changes in nucleotide sequences observed in BVDV-infected goats might be explained as a strategy for viral transmission to infect a new host. Consequently, these differences in the sequence may be essential to the process of establishing a BVDV infection in a new host. It has been reported that RN`11A viruses have a tendency to change hosts more frequently than other pathogens [24–26]. The virus may be pre-adapted to a new host, and these nucleotide changes may enhance the ability of the virus to establish infection in a new host. Bachofen et al. evaluated interspecific transmission from cattle to goats but did not identify any nucleotide changes in 5′-UTR [18]. However, changes were observed in the E2-coding region, which were quite different from our results. Although we did not evaluate nucleotide differences in the E2 region in this study, we found genetic changes in Korean native goats after BVDV infection in both the 5′-UTR and N^pro^ regions; however, genetic changes were highly significant in the N^pro^ region. Nucleotide changes may be necessary for the transmission of BVDV infection from cattle to goats. These results suggest that Korean native goats may possibly act as a reservoir host of BVDV.

## Conclusions

Our results show that BVDV can establish an infection in Korean native goats and exhibits minimal clinical symptoms. The observed genetic changes may be explained as a result of the establishment of BVDV infection in a new host, and these consequences were found to be host-dependent. The shedding of BVDV from infected animals may facilitate the maintenance and transmission of BVDV to other hosts. Based on our findings, it is likely that Korean native goats play a role in BVDV maintenance in the ROK. Therefore, understanding interspecific transmission may be important for BVDV eradication programs.

## Methods

### Ethics statement

Korean native goats used in this study were acquired from a private farm located in Mungyeong, ROK. All goats were sacrificed by a veterinarian in accordance with the guidelines of the Animal Ethics Committee of Kyungpook National University (approval number: 2016–00040).

### Virus preparation

The noncytopathic (ncp) BVDV-1b strain 11Q472 was isolated from a 2-year-old Korean indigenous cow presenting with emaciation, loss of appetite, and diarrhea in the ROK [23]. The virus strain was obtained after culturing for 5 days with one freeze-thaw cycle and centrifuging at 1900×*g* for 10 min to remove large cellular debris. The supernatant was aliquoted and then frozen at − 80 °C until inoculation. Viruses in the 5th passage of Madin-Darby Bovine Kidney (MDBK) cells were selected for inoculation; they were titrated from the MDBK cell cultures, and the median tissue culture-infective dose (TCID_50_) was calculated. MDBK cells were confirmed to be free of mycoplasma contamination using PCR (Takara Bio Inc., Japan). The MDBK cells were maintained in minimum essential medium (MEM; Life Technologies Corp., Carlsbad, CA, USA) supplemented with 5% horse serum (Thermo Fisher Scientific Inc., Waltham, MA, USA).

### Experimental animals and virus infection

Korean native goats were maintained under pathogen-free conditions and handled in strictly in accordance with the guidelines and protocols approved by the Kyungpook National University Animal Care and Use Committee (approval number: 2016–00040). Goats used for this study were 3-month-old healthy females. The goats were negative for the BVDV antibody and antigen, as determined by the enzyme-linked immunosorbent assay and RT-PCR. Four goats were infected with 2.5 × 10^6^ TCID_50_ of ncp BVDV-1b via IN route, and two goats served as uninfected controls (mock-infected) and received MEM (Life Technologies). The goats were housed individually. All experiments were conducted twice.

### Sample collection, RT-PCR, and sequence analysis

Goats were monitored daily during the experiment. The body temperature was measured and recorded every other day at approximately the same time. Nasal swabs and blood samples were collected on 0, 2, 5, 7, 9, 12, 14, 16, 19, and 21 dpi, and the animals were euthanized on 21 dpi. Blood samples were obtained in Vacutainer tubes containing ethylenediaminetetraacetic acid (Becton Dickinson, Franklin Lakes, NJ, USA). RNA was extracted from the nasal swabs and blood samples using the PureLink® Total RNA Blood Kit (Invitrogen, Carlsbad, CA, USA). RT-PCR was performed using primers designed to amplify the 5′-UTR and N^pro^ region (Table 3). The PCR products were purified using the AccuPrep® PCR Purification Kit (Bioneer, Daejeon, Korea), and the amplicons were used for direct sequencing (Bioneer). The sequence data were analyzed using Chromas software (version 2.33, http://www.technelysium.com.au/chromas.html), and alignments were obtained using CLUSTAL X (version 1.8). The amplified sequences were compared with those of the virus used for experimental inoculations. A phylogenetic tree was constructed based on nucleotide alignments using the neighbor-joining method [27]. A bootstrap analysis was performed with 1000 replicates using MEGA software version 6 [28].

**Table 3:** Primers used to detect BVDV in this study

Target gene	Sequence (5′-3′)	*Tm* (°C)/Time	Amplicon size
5′-UTR	F, ATG CCC WTA GTA GGA CTA GCA	55 °C, 40 s	288 bp
	R,TCA ACT CCA TGT GCC ATG TAC		
N^pro^	F, TCT CTG CTG TAC ATG GCA CAT G	52 °C, 1 min	425 bp
	R,CCA TCT ATR CAC ACA TAA ATG TGG T		

### Enzyme-linked immunosorbent assay

Antibody detection was performed using serum samples from the ncp BVDV-1b-infected and mock-infected goats with SVANOVIR® BVDV p80-Ab (SVANOVA, Uppsala, Sweden) according to the manufacturer’s instructions. Serum samples were added to a 96-well plate which was pre-coated with BVDV antigen, and subsequently incubated them for 1 h at 37 °C. Plates were washed, supplemented with horseradish peroxidase-labeled conjugate, and incubated for 1 h at 37 °C. After washing, the substrate solution was added and incubated for 10 min, followed by the addition of stop solution. The optical density was measured at 450 nm using a microplate reader (Tecan, Mannedorf, Switzerland). Samples were classified as positive when inhibition percentage was ≥45%.

## Additional files

Additional file 1: **Table S1.** BVDV isolates used in the phylogenetic tree based on the 5′-UTR. (DOCX 17 kb)

Additional file 2: **Table S2.** BVDV isolates used in the phylogenetic tree based on the Npro region. (DOCX 18 kb)

## Data Availability

The datasets used and analyzed during the current study are available from the corresponding author on reasonable request.

## Abbreviations

5′-UTR: 5′- untranslated region
BDV: Border disease virus
BVDV: Bovine viral diarrhea virus
CSFV: Classical swine fever virus
dpi: Days post-infection
IN: Intranasal
ncp: Noncytopathic
N^pro^: N-terminal protease
ROK: Republic of Korea
RT-PCR: Reverse transcription-polymerase chain reaction
TCID_50_: Median tissue culture infective dose
