# Supplementary material for: Experimental infection of Korean native goats (Capra aegagrus hircus) with bovine viral diarrhea virus 1b
Source: BMC Vet Res. 2019 Jun 14;15:202. doi: 10.1186/s12917-019-1955-0 (PMC6570889; doi:10.1186/s12917-019-1955-0)
Supplement: Supplementary file 1 — Table S1. BVDV isolates used in the phylogenetic tree based on the 5′-UTR. (DOCX 17 kb) [file 12917_2019_1955_MOESM1_ESM.docx]

Additional file 1: Table S1. BVDV isolates used in the phylogenetic tree based on the 5′-UTR

| **Accession number** | **Host** | **Subgenotype** | **Country** |
| --- | --- | --- | --- |
| EF406123 | Cattle | 1b | Brazil |
| GQ495691 | Cattle | 1b | South Korea |
| GU395543 | Cattle | 1b | USA |
| JN542508 | Cattle | 1b | China |
| JQ646075 | Cattle | 1b | Argentina |
| KF205314 | Cattle | 1b | France |
| KX280711 | Calf | 1b | China |
| KY499141 | Cattle | 1b | South Korea |
| MF120594 | Cattle | 1b | Argentina |
| MG434595 | Cattle | 1b | Italy |
| MH753467 | Cattle | 1b | Turkey |
| JN715035* | Cattle | 1b | Poland |
| GU395535* | Cattle | 1b | USA |

*These were used in Figure 2.
