# Supplementary material for: Experimental infection of Korean native goats (Capra aegagrus hircus) with bovine viral diarrhea virus 1b
Source: BMC Vet Res. 2019 Jun 14;15:202. doi: 10.1186/s12917-019-1955-0 (PMC6570889; doi:10.1186/s12917-019-1955-0)
Supplement: Supplementary file 2 — Table S2. BVDV isolates used in the phylogenetic tree based on the Npro region. (DOCX 18 kb) [file 12917_2019_1955_MOESM2_ESM.docx]

Additional file 2: Table S2. BVDV isolates used in the phylogenetic tree based on the Npro region

| **Accession number** | **Host** | **Subgenotype** | **Country** |
| --- | --- | --- | --- |
| KF023470 | Cattle | 1b | UK |
| KF205332 | Cattle | 1b | France |
| KP127974 | Cattle | 1b | Egypt |
| KX853086 | Cattle | 1b | Poland |
| KF205321 | Cattle | 1b | France |
| KF023381 | Cattle | 1b | UK |
| JN542498 | Cattle | 1b | China |
| JN833764 | Cattle | 1b | Poland |
| AJ585412 | Cattle | 1b | Hungary |
| EU180035 | Cattle | 1b | Switzerland |
| JN704144 | Cattle | 1b | China |
| JX088006 | Cattle | 1b | China |
| KC695814 | Cattle | 1b | USA |
| KC963967 | Cattle | 1b | South Korea |
| KF772785 | Cattle | 1b | China |
| KP941583 | Calf | 1b | USA |
| KP941587 | Calf | 1b | USA |
| KP941589 | Calf | 1b | USA |
| KP941590 | Calf | 1b | USA |
| KR029825 | Cattle | 1b | Egypt |
| EF101530* | Cattle | 1b | Germany |
| U03912* | Cattle | 1b | Germany |

*These were used in Figure 3.
